# Splenic monocytes drive pathogenic subretinal inflammation in age-related macular degeneration

**DOI:** 10.1186/s12974-024-03011-z

**Published:** 2024-01-17

**Authors:** Christophe Roubeix, Caroline Nous, Sébastien Augustin, Kaitryn E. Ronning, Thibaud Mathis, Frédéric Blond, Pauline Lagouge-Roussey, Sergio Crespo-Garcia, Patrick M. Sullivan, Emmanuel L. Gautier, Nadine Reichhart, José-Alain Sahel, Marie E. Burns, Michel Paques, Torben Lykke Sørensen, Olaf Strauss, Xavier Guillonneau, Cécile Delarasse, Florian Sennlaub

**Affiliations:** 1 Sorbonne Université, INSERM, CNRS, UMR_S 968, Institut de la Vision, 75012 Paris, France; 2 Charité - Universitätsmedizin Berlin, corporate member of Freie Universität Berlin and Humboldt Universität zu Berlin, Experimental Ophthalmology, Department of Ophthalmology, Charitéplatz 1, 10117 Berlin, Germany; 3 Service d’Ophtalmologie, Centre Hospitalier Universitaire de la Croix-Rousse, Hospices Civils de Lyon, Université Claude Bernard Lyon 1, 69004 Lyon, France; 4 Department of Medicine, Centers for Aging and Geriatric Research Education and Clinical Center, Durham Veteran Affairs Medical Center, Duke University, Durham, NC 27710 USA; 5 Sorbonne Université, INSERM, UMR_S 1166, Hôpital de la Pitié-Salpêtrière, 75013 Paris, France; 6 Center for Neuroscience, Department of Cell Biology and Human Anatomy, Department of Ophthalmology and Vision Science, University of California, Davis, CA 95616 USA; 7 Centre Hospitalier National d’Ophtalmologie des Quinze-Vingts, INSERM-DHOS Clinical Investigation Center 1423, Paris, France; 8 Clinical Eye Research Division, Department of Ophthalmology, Zealand University Hospital Roskilde, Roskilde, Denmark; 9 Faculty of Health and Medical Science, University of Copenhagen, Copenhagen, Denmark

**Keywords:** Age-related macular degeneration, Angiotensin, Neuroinflammation, Splenic monocytes

## Abstract

Age-related macular degeneration (AMD) is invariably associated with the chronic accumulation of activated mononuclear phagocytes in the subretinal space. The mononuclear phagocytes are composed of microglial cells but also of monocyte-derived cells, which promote photoreceptor degeneration and choroidal neovascularization. Infiltrating blood monocytes can originate directly from bone marrow, but also from a splenic reservoir, where bone marrow monocytes develop into angiotensin II receptor (ATR1)^+^ splenic monocytes. The involvement of splenic monocytes in neurodegenerative diseases such as AMD is not well understood. Using acute inflammatory and well-phenotyped AMD models, we demonstrate that angiotensin II mobilizes ATR1^+^ splenic monocytes, which we show are defined by a transcriptional signature using single-cell RNA sequencing and differ functionally from bone marrow monocytes. Splenic monocytes participate in the chorio-retinal infiltration and their inhibition by ATR1 antagonist and splenectomy reduces the subretinal mononuclear phagocyte accumulation and pathological choroidal neovascularization formation. In aged AMD-risk ApoE2-expressing mice, a chronic AMD model, ATR1 antagonist and splenectomy also inhibit the chronic retinal inflammation and associated cone degeneration that characterizes these mice. Our observation of elevated levels of plasma angiotensin II in AMD patients, suggests that similar events take place in clinical disease and argue for the therapeutic potential of ATR1 antagonists to inhibit splenic monocytes for the treatment of blinding AMD.

**Supplementary Information:**

The online version contains supplementary material available at 10.1186/s12974-024-03011-z.

## Introduction

Age-related macular degeneration (AMD) is a common, highly heritable, neuroinflammatory disorder defined by subretinal deposits (drusen) in its early form and choroidal neovascularization (wet AMD) or an extending area of atrophy (geographic atrophy) in its late form [1, 2]. The early and both late forms are characterized by the activation and the chronic accumulation of subretinal mononuclear phagocytes (MPs), a family of cells that include monocytes, and infiltrating- and resident-macrophages such as microglial cells [1]. Under physiological conditions, the photoreceptor cell layer and subretinal space is immunosuppressive and devoid of blood- and lymphatic vessels, as well as any MPs, including microglial cells [1]. We recently demonstrated that the two main genetic AMD-risk variants, CFH Y402H and a haplotype of 10q26, directly promote the accumulation of pathogenic subretinal MPs, emphasizing the role of MPs and inflammation in AMD [3, 4]. The MP infiltrate in AMD is not only composed of displaced microglial cells (the resident MP of the retina) [5], but also of MPs derived from infiltrating monocytes (Mo) [1, 6, 7]. Moreover, in patients with neovascular- [8–11] and atrophic-AMD [6, 12] the intraocular concentration of the monocyte-attractants such as CCL2 is increased. Experimentally, Mo-derived cells (MdCs), macrophages and dendritic cells, represent 20–50% of the subretinal MPs during the early inflammatory reaction [6, 13, 14] and promote photoreceptor degeneration [6, 15–22] and choroidal neovascularization [23–27] in a range of mouse models (reviewed in [1, 28]).

Mos differentiate as CCR2^+^Ly6C^high^Mos from hematopoietic stem cells in the bone marrow (BM) [29, 30], which convert into intermediate-, and then non-classical CCR2^neg^Ly6C^low^Mos in the blood over a time period of around seven days [31, 32]. However, an important number of “classical” CCR2^+^Ly6C^high^Mos also join a splenic reservoir, where they convert into angiotensin II (ANGII) receptor (ATR1)^+^ splenic Ly6C^high^Mos (spleMos). Remarkably, the ATR1^+^Ly6C^high^spleMos pool outnumbers the blood counterparts [33]. Under pathological conditions not only the blood Ly6C^high^Mos [34, 35], but also the ATR1^+^Ly6C^high^spleMos [33, 36] are recruited into the diseased tissue and differentiate into “inflammatory” MdCs. Reservoir spleMo have been shown to be of particular pathogenic importance in atherosclerosis [36], and Tan et al. reported a similar pathogenic role of spleMos in the laser model of wet AMD [37]. These results demonstrated the potential importance of ATR1^+^Ly6C^high^spleMos.

Angiotensin II (ANGII), a vasoconstricting peptide that acts as a key regulator of blood pressure and sodium retention by the kidney, has been shown to mobilize and recruit ATR1^+^Ly6C^high^spleMos to myocardial infarction [33, 38]. However, ANGIIs influence on spleMo mobilization in conditions such as AMD and most other scenarios remains unexplored. ANGII is produced by the activation of the renin–angiotensin system (RAS). Its chronic increase leads to hypertension, a risk factor for late AMD [39]. Although there is no significant clinical association of AMD prevalence with RAS inhibitor usage per se, AMD prevalence is inversely correlated with RAS inhibitor treatment duration [40]. In laser- and light-induced AMD models, ANGII receptor (ATR1) inhibition has been shown to reduce tissue damage and neovascularisation [41, 42] and visual impairment in mice under high-fat diet [43]. The beneficial effect of the ATR1 inhibition has been primarily attributed to vascular ATR1 inhibition and reduction of lipid accumulation in resident macrophages induced by high-fat diet. The potential implication of ATR1^+^Ly6C^high^spleMos has not been explored in these studies.

Using various pathogenic subretinal inflammatory models, we here demonstrate that ANGII mobilizes ATR1^+^Ly6C^high^spleMos and that they play an important pathological role in both acute (laser- or light-induced) and chronic models of subretinal inflammation. We further show that spleMos differ transcriptionally and functionally from BM-derived Mos (BMMos). ATR1 antagonist treatment with losartan decreases the pathogenic subretinal inflammation in acute and chronic models. Our results showing an association of elevated plasma concentrations of ANGII with AMD suggest that similar mechanisms underlie the human disease. Together our results argue for the therapeutic potential of spleMo inhibition for the treatment of advanced AMD.

## Material and methods

*Animals* 7-week-old C57BL/6J wild-type mice were purchased from the Janvier Breeding Center (Le Genest-St-Isle, France). Wild-type and *Rag2*^*−/−*^ mice were purchased (Charles River Laboratories, Jackson laboratories), and targeted replacement mice that express human APOE2 isoforms (*TRE2*) were engineered as previously described [44]. *Rag2*^*−/−*^ and *TRE2* mice were bred and maintained in the animal facility. All mice were negative for the *Crb1*^*rd8*^, *Pde6b*^*rd1*^, and *Gnat2*^*cpfl3*^ mutations. Mice were housed in the animal facility under specific pathogen-free conditions, in a 12/12 h light/dark (100–500 lx) cycle with water and normal diet food available ad libitum. They were acclimatized for 1 week before experimentation.

*In vivo laser-injury and light-challenge experiments* Laser photocoagulation was performed on male C57BL/6J mice at the indicated ages (Vitra Laser, 532 nm, 450 mW, 50 ms and 250 mm). The pupils of the mice were fully dilated with mydriaticum and neosynephrine, and animals were anesthetized with a solution of ketamine (80 mg/kg) and xylazine (8 mg/kg). Laser burns are induced at the 3, 6, 9 and 12 o’clock positions in the mid-periphery. Lubrithal was placed on the eyes while the animals recovered from the anesthesia to maintain ocular surface moisture. The mice were killed and eyes were enucleated and processed for immunohistochemistry analysis at the indicated time points.

*Light-challenge model* *TRE2* mice were dark adapted for 6 h and their pupils were fully dilated with 1% atropine (Novartis) each day of light exposure. The animals were exposed to green LED light (4500 Lux, JP Vezon equipment) for 4 days. MP accumulation was assessed immediately after the fourth day of light exposure: eyes were enucleated and processed for immunohistochemistry analysis.

*ANGII intraperitoneal injection* 7-week-old C57BL/6J wild-type mice were injected intraperitoneally with 100 µl of PBS or 100 µl PBS containing ANGII (Sigma-Aldrich) for a final dose of 1.2 mg ANGII/kg. This dose was chosen to evaluate the maximum effect that can be expected from circulating ANGII, which roughly corresponds to a daily dose of the ANGII pumps (see below). Spleen and BM were sampled 3 h after the injection for weighing and to be processed for flow cytometry.

*ANGII pump* Minipump (Model 2004; ALZA Corp., Palo Alto, CA) were subcutaneously transplanted in 8- to 10-week-old C57BL/6J mice previously filled with PBS or ANGII (1 µg/kg/min for 14 days (based on the dosage used by Swirski et al. 2009 [33]) Angiotensin II human A9525; Sigma Aldrich). Pumps were primed 24 h before transplantation in a NaCl 0.9% solution overnight at 37 °C following manufacturer recommendations.

*Losartan treatment* The day before laser-induced CNV or light exposure, mice were injected intraperitoneally with losartan (10 mg/kg, 10006594-Cayman chemical) and then injected daily until the day of killing. Intraperitoneal losartan dosages were based two previous reports of laser-induced CNV and light-induced neural damage in the retina [41, 42]. Control mice were injected with the same volume of PBS. 12-month-old *TRE2* mice were treated for 3 months with losartan diluted in the drinking water. An approximation of 5 ml of daily water consumption per mouse and an estimated bioavailability of 30% of the orally administered dose were used to calculate a 30 mg/kg/day intake of losartan per mice per day. This dose corresponds to the average dose found in the literature to assess the cardiovascular effect of the drug [45].

*Splenectomy* 30 min before intervention, a subcutaneous injection of buprenorphine (0.05 mg/kg) was administered. Mice were anesthetized with ketamine (80 mg/kg) and xylazine (8 mg/kg) solution and a 1-cm incision was made on the left side of the abdominal cavity under the rib cage. Mice were randomly selected for a splenectomy or sham procedure. The spleen was removed by cutting the mesentery and connective tissue and the splenic vessels were cauterized. For sham-control mice, incisions were made without removing the spleen. A buprenorphine (0.05 mg/kg) injection was administered postoperatively and at one day after intervention.

*Reverse transcription and real-time polymerase chain reaction* Total RNA was isolated with Nucleospin RNAII (Macherey Nagel). Single‐stranded cDNA was synthesized from total RNA (pre-treated with DNaseI amplification grade) using oligo‐dT as primer and superscript II reverse transcriptase (Life technologies). Subsequent RT‐PCR was performed using cDNA, a Taqman Rps26 assay and the Agtra1 Taqman assay Mm01957722_s1 that recognize sequences in exon 3 that are shared in all known Agtra1 isoforms. Results were normalized by expression of rps26. PCR reactions were performed in 45 cycles of 15 s at 95 °C, 45 s at 60 °C.

*RNAscope* Spleen cryosections were fixed for 1 min in 4% PFA at room temperature, then washed 3 times in PBS and dehydrated in successive 50, 70, and 100% ethanol baths. Staining was then performed according to the manufacturer’s instructions using the primers for *Agtr1a* and *Itgam* (Hi-Plex protocol, Bio-Techne). After mounting, the slides were observed with a fluorescence microscope (DM5500, Leica).

*Immunohistochemistry on spleen cryosections* Fresh spleens were embedded in OCT and frozen using liquid nitrogen. Then they were cut with cryostat at − 20 °C (CM3050S, Leica); section 10 µm. Spleen cryosections were fixed for 15 min in 4% PFA at RT, then incubated with blocking solution (PBS-0.1% triton 5% Horse Serum (HS)) for 1 h at RT. After they were incubated overnight at 4 °C in PBS-0.1% Triton 0.8% HS with conjugated antibody Ly6C-FITC (Miltenyi; 1/100) and nuclei were counterstained with Hoechst (Sigma Aldrich; 1/1000). After mounting, they were viewed with a fluorescence microscope (DM5500, Leica).

*Immunohistochemistry on RPE/choroid/retina* Eyes were fixed for 30 min in 4% PFA at room temperature (RT) before dissection and were sectioned at the limbus; the cornea and lens were discarded. The retinas were carefully peeled from the RPE/choroid/sclera and incubated overnight at 4 °C in PBS-1% triton with the following primary antibodies:

- Peanut agglutinin (PNA) Alexa Fluor 594 (Thermo Fisher Scientific; 1/100) and rabbit polyclonal antibody anti-IBA1 (Wako pure chemical industries; Osaka, Japan; 1/400) for light challenge model.
- Rat anti-mouse CD102 (clone 3C4, BD Biosciences Pharmingen; 1/400), goat polyclonal anti-collagen IV antibody (Bio-Rad; 1/400) and rabbit polyclonal antibody anti IBA1 (Wako pure chemical industries; Osaka, Japan; 1/400) for laser-induced CNV model.

After a few washes, retinas and RPE/choroid/sclera complexes were incubated for 2 h at RT with appropriate Alexa Fluor^®^ conjugated secondary antibodies (Thermo Fisher Scientific; 1/500) in PBS and nuclei were counterstained with Hoechst (Sigma Aldrich; 1/1000). They were flatmounted and viewed with a fluorescence microscope (DM5500, Leica). The laser impact was captured in a single‐plane image. Subretinal, perilesional MPs were counted on the RPE surrounding each laser burn at a distance from 0 to 500 µm. The area of the lesion was measured using ImageJ software. PNA^+^ cone cell counts are the average of cone density in all quadrants quantified on 40 × photographs taken of the mid-periphery of stained retinal flatmounts. For all experiments the n corresponds to the average of the measurements from one experimental animal.

*Flow cytometry* Preparation of cell suspensions was performed as follows:

- Blood was collected with a glass capillary from the suborbital veins at the time of killing. 200 µl of blood was collected from each mouse and the red blood cells were lysed using red blood cell lysis buffer (BD Biosciences) prior to immunostaining of 50 µl of the leukocyte suspension.
- Spleens were mechanically filtered on a 70-µm cell strainer and rinsed with PBS to obtain a 10 ml cell suspension. The tubes were then centrifuged (500 g, 5 min). The red blood cells were lysed using ammonium chloride (StemCell Technologies) and resuspended in 2.5 ml of PBS. Immunostaining was then performed on 50 µl of the cell suspension.
- One femoral bone per mouse was removed and cleaned. The epiphyses were sectioned and the BM was rinsed with 2 ml of PBS. After centrifugation (500 g, 5 min), the cells were resuspended in 2 ml of PBS and 50 µl of the cell suspension was collected to perform immunostaining.
- Individual eyes were collected on ice and dissected by first sectioning the limbus and removing the cornea and lens. The eye cups were incubated at 37 °C for 30 min in PBS containing the enzyme Liberase TL at 1.6 Wunsch unit/ml (Sigma-Aldrich). Individual eye cups were filtered onto a 70-µm cell strainer and rinsed with PBS to obtain a 10 ml cell suspension. The tubes were then centrifuged (500*g*, 5 min) and resuspended in 100 µl PBS.

Cytometry was performed using the following antibody cocktail for Ly6C^high^Mo quantification: anti-CD45 Vioblue, anti-CD11b PE, anti-Ly6C PE-Vio770, and anti-Ly6G APC-Vio770 (all from Miltenyi). Dead cells were excluded from the analysis using a Live/Dead fixable VioGreen staining reagent (Miltenyi). Acquisition was performed on the Celesta SORP cytometer (BD Biosciences), and data were analyzed with FlowJo 10.8.

Cell-sorting of Ly6C^high^Mo from mouse RPE/choroid/retina, spleen and bone-marrow for scRNAseq: preparation of cell suspensions was performed as described for flow cytometry.

Spleen and BM cells from five C57BL/6J mice were labeled with the following antibodies: anti-CD45 Vioblue, anti-CD11b PE, anti-Ly6C PE-Vio770, anti-Ly6G APC-Vio770. Dead cells were excluded from the analysis using a Live/Dead fixable VioGreen staining reagent (Miltenyi). 1 × 10^5^ CD45^+^CD11b^+^Ly6C^high^Ly6G^neg^ Mos were sorted and collected in a cold tube and then used for scRNA-sequencing (below).

All cell suspensions from the eyes of 10 splenectomized or sham-operated mice were pooled and stained with the following primary antibodies: anti-CD45 VioBlue, anti-CD11b PE, anti-Ly6C PE-Vio770, anti-Ly6G FITC. Dead cells were excluded from the analysis using a Live/Dead fixable VioGreen staining reagent (Miltenyi). All CD45^+^CD11b^+^Ly6G^neg^ MPs were then sorted and collected for each group, and then used for scRNA-sequencing (below).

### Single-cell RNA sequencing

The barcoded cDNAs were prepared from sorted cells using a 10 × genomics chromium and were used to construct the indexed scRNA-sequencing libraries according to Chromium Next GEM Single Cell 3′ Library v3.1 and dual Index Kit TT according to manufacturer’s protocol. Libraries were sequenced on an Illumina Novaseq 6000 sequencer. The Fastq files were processed using Cellranger v6.1.2 with default options (alignment, count). The resulting expression matrices were merged and analyzed with Seurat v4 (merge, QC filtering, normalization, PCA, clustering, UMAP/tSNE). The pseudotime analysis was performed using the Monocle3 package. The results were added into the previous Seurat object to create a H5AD file that was used as input for cellxgene for visualization and data exploration. Further analyses were performed in cellxgene using the cellxgene_VIP plugin (differential analysis, various visualizations, …). Processing scripts are available on demand. Gene ontology (GO) analyses for functional enrichments were performed using DAVID bioinformatics resources [46] and STRING consortium [47].

Enriching murine bone marrow and spleen monocytes: Mice BMMos and mice spleen monocytes (SpleMos) were harvested from 2- to 3-month-old male C57BL/6J mice killed by CO2 inhalation. BMMos were flushed from femurs and tibia with PBS containing 5% fetal bovine serum while spleens were passed through cell strainers rinsed with PBS to obtain a cell suspension. BMMos and SpleMos were negatively selected by magnetic sorting following the protocol suggested by the manufacturer (EasySep Mouse Monocyte Enrichment Kit; StemCell Technologies, Inc.). Briefly, the mouse Mo enrichment mixture is designed to enrich mouse Mos from mouse by depletion of T cells, B cells, NK cells, dendritic cells, progenitors, granulocytes, and red blood cells using a combination of biotinylated monoclonal antibodies directed against cell-surface antigens. Unwanted cells were specifically labeled with dextran-coated magnetic particles using biotinylated antibodies against cell-surface antigens expressed on the unwanted cells. Magnetically labeled cells were then separated from unlabeled target cells by using a magnet. The purity of the Mos was assayed by flow cytometry using CD11b, Ly6C, and Ly6G as markers. The CD11b^+^Ly6C^+^Ly6G^−^ (Mos) cell content of the enriched cells ranges from 85 to 95%. The rest of the enriched cells were CD11b^+^Ly6G^+^ cells (neutrophils).

Subretinal adoptive mononuclear phagocyte transfer and clearance: BMMos and SpleMos were isolated as described above, labeled in 10 mM CFSE (Thermo Fisher Scientific), washed and resuspended in PBS. 12,000 cells (in 4 µL) were injected in the subretinal space of anesthetized WT male mice (10–14 weeks old) using glass microcapillaries (Eppendorf) and a microinjector as described before [3, 4, 48]. Briefly, first a hole was pierced with the glass capillary through the sclera and retina into the vitreous to allow efflux of vitreous and decompression. The glass capillary is than pierced through a second site and advanced to the subretinal space opposite the decompression hole, where 4 µl of solution is injected. The subretinal injection is verified by fundoscopy and in the rare event that we failed to induce a retinal detachment or subretinal- or vitreal-hemorrhages are induced the eyes are discarded from further evaluation. This procedure leads to a transient retinal detachment of 30–50% without inducing retinal tears, and the subretinal fluid is quickly resorbed and not detected anymore after 24 h [3, 4, 48]. Eyes were enucleated after 24 h, fixed 30 min in PFA 4% and counterstained with Hoechst nuclear stain. Eyes with hemorrhages were discarded. CFSE^+^ cells in the subretinal space were quantified on flatmounts on the RPE side of the retina and on the apical side of the RPE.

Plasma RAS fingerprint: Frozen plasma from patients and mice were used to measure equilibrium ANGI and ANGII concentrations (eqANGI and eqANGII, respectively) and to assess plasma renin activity (PRA). We measured eqANGI and eqANGII instead of direct plasma measurements, because of the rapid speed at which RAS peptides are degraded. Samples were sent to Attoquant Diagnostics (Vienna, Austria) where they were processed as follows: samples were incubated at 37 °C and pH 7.4 for 1 h to allow ANGI and ANGII production and degradation to reach a stable equilibrium in the sample, followed by LC–MS/MS. As blood angiotensinogen (ANG) is in a very large “molar excess” (micromolar range) compared with renin and ACE (picomolar range) these “equilibrium” concentrations are used as an approximation of the in vivo RAS activity, ANGI, and ANGII concentrations [49]. Equilibrium of the RAS metabolites was rapidly reached and stable for several hours. Samples were also sent to Attoquant Diagnostics to determine PRA using the AGTI radioimmunoassay kit (Immunotech) as follows: samples were diluted in an ANGI stabilizing buffer containing additional ANG before a 1 h incubation at 37 °C, and the generated ANGI was determined as a measure of plasma renin activity [(ng ANGI/ml)/h] [49].

Data analysis: Sample sizes for our experiments were determined according to our previous studies. Graph Pad Prism 9 (GraphPad Software) was used for data analysis and graphic representation. All values are reported as mean ± SEM. Statistical analysis was performed by Mann–Whitney U-test for 2-group comparisons, and one-way ANOVA followed by a Brown–Forsythe and Welch test for data with normal distribution or by Kruskal–Wallis test if this was not the case. For all laser experiments, the n corresponds to the average calculated from the measurements of one experimental animal. The n and p-values are indicated in the figure legends.

## Results

### ANGII mobilizes spleMos and promotes subretinal inflammation spleen dependently

ANGII mediates most of its effects through ATR1 encoded by the *Agtr1a* gene. The beneficial effects of ATR1 inhibition in a high-fat diet model of AMD have been attributed to ATR1 inhibition on choroidal macrophages [43] and to vascular ATR1 inhibition in laser-induced choroidal neovascularization (CNV), a model of wet AMD [42]. Using the same model of laser-induced CNV, Tan et al*.* showed an important role of the spleen in the local macrophage recruitment [37], but whether and to what degree ANGII-dependent spleMos mobilization plays a role is unknown.

**Fig. 1:**
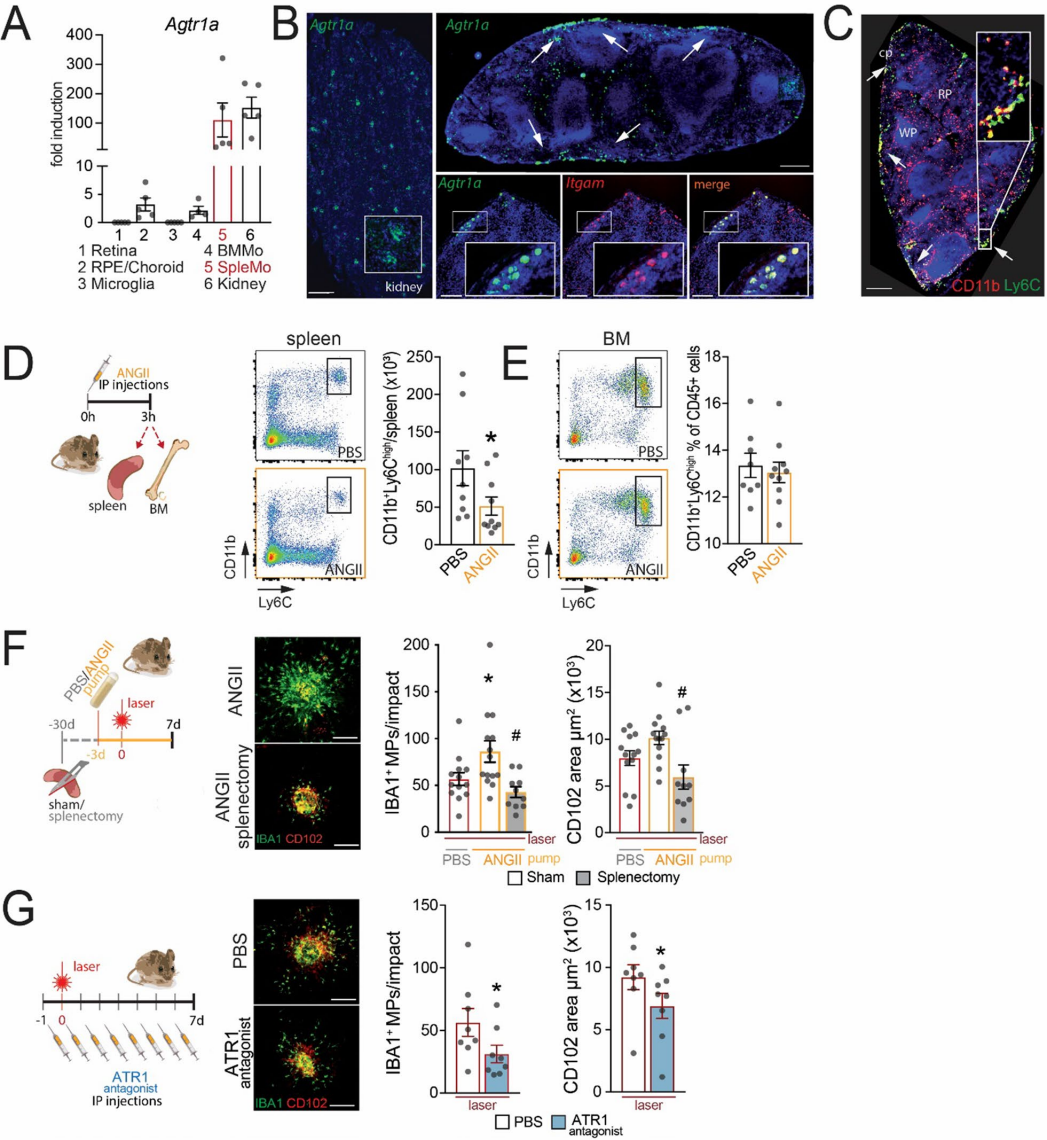
ANGII mobilizes spleMos and promotes subretinal inflammation spleen dependently. **A** RT-PCR results of *Agtr1a* mRNA normalized with *Rps26* mRNA of fresh FACS-sorted Ly6C^high^ bone marrow monocytes (BMMos), Ly6C^high^ spleen monocytes (SpleMos), and retinal microglial cells, as well as freshly homogenized retina, RPE/choroid, and kidney (*n* = 5, samples from three independent experiments). **B** Representative image of an RNAscope in situ hybridization of *Itgam* mRNA (CD11b) (red) and *Agtr1a* mRNA (green) of a kidney (positive control) and spleen cryosection. Three independent experiments gave similar results. Scale bar = 1 mm. **C** Representative image of a CD11b (red) and Ly6C (green) double-immuno-labeled spleen cryosection. Scale bar = 1 mm. **D**, **E** Quantification by flow cytometry of CD11b^+^Ly6C^high^ cells in the spleen (**D**) and in the BM (**E**) 3 h after PBS or ANGII intraperitoneal injections (*n* = 10; Mann–Whitney test, **D** PBS vs ANGII: *p = 0.0434). **F** Representative images of IBA1 (green, MP marker) and CD102 (red) immunostained RPE flatmounts 7 days after laser-injury of sham-operated or splenectomized (− 30 days) mice that were transplanted with subcutaneous pumps (− 3 day) filled with PBS or ANGII (1 µg/kg/min), as indicated in the cartoon. Quantification of IBA1^+^ MPs surrounding laser impacts and CD102^+^ CNV area (*n* = 5–6; one-way ANOVA, PBS Sham vs ANGII Sham: MPs *p = 0.0495; ANGII Sham vs ANGII splenectomy: MPs ^#^*p* = 0.0023, CD102^+^ area ^#^*p* = 0.0124). Scale bar = 20 µm. **G** Representative images of IBA1 (green) and CD102 (red) immunostained RPE flatmounts 7 days after laser impact of mice treated by intraperitoneal injection of PBS or ATR1 antagonist losartan (10 mg/kg) as indicated. Quantification of IBA1^+^ MPs surrounding laser impacts and CD102^+^ CNV area of these mice. (*n* = 8; Mann–Whitney test, PBS vs Losartan, MPs *p = 0.0172; CD102^+^ area *p < 0.0001). Scale bar = 20 µm. RPE: retinal pigment epithelium; BMMo: bone marrow monocyte; spleMo: splenic monocyte; IP injection: intraperitoneal injection; BM: bone marrow; ANGII: angiotensin II; ATR1: angiotensin II receptor type 1

To evaluate which cells likely respond to ANGII, we first compared *Agtr1a* transcription in ocular tissue, microglial cells and Mos from the BM and spleen. Although our primers recognize all known *Agtr1a* splice variants, we did not detect significant *Agtr1a* transcription in bulk retina, contrary to previous reports in rats [50] or in FACS-sorted retinal microglia under steady state. This discrepancy might be due to species differences of expression levels. We found *Agtr1a* mRNA to be weakly expressed in the choroid and sorted BMMo compared to much stronger transcription in FACS-sorted spleMo, which revealed high expression levels comparable to the kidney positive control (Fig. 1A).

As ATR1 immunohistochemistry is notoriously unreliable [51], we next localized *Agtr1a* mRNA by in situ hybridization. Hybridization of kidney and spleen sections confirmed *Agtr1a* mRNA localization to the glomeruli (positive control) and demonstrated their strong expression in the spleen in subcapsular round cells that also transcribed *Cd11b* mRNA (Fig. 1B). Immunohistochemical double labeling of CD11b and Ly6C confirmed the subcapsular CD11b^+^ cells identity as Ly6C^high^spleMo (Fig. 1C) in agreement with the high *Agtr1a* mRNA expression detected in FACS-sorted Ly6C^high^spleMos (Fig. 1A).

To evaluate the effect of ANGII on spleMo and BMMo, we next injected a single dose of ANGII intraperitoneally. After 3 h ANGII reduced the numbers of CD11b^+^Ly6G^−^Ly6C^high^spleMos per spleen evaluated by FACS in comparison to PBS (Fig. 1D), while CD11b^+^Ly6G^−^Ly6C^high^BMMos were unchanged (Fig. 1E).

Next, we tested the effect of exogenous ANGII and spleMos on MP accumulation and persistence in the immunosuppressive subretinal space in the laser-induced CNV models. The laser-injury induces the infiltration of subretinal MPs, with a maximal recruitment three to four days after the injury and a maximal neovascular response seven to ten days after the injury [52]. The infiltrating MPs are observed in great numbers within the within the laser-induced lesion, where the immunosuppressive RPE is lacking. However, they are also observed surrounding the lesion where they accumulate on the immunosuppressive RPE cells [3, 48, 52–54]. While the density of MPs in the RPE denuded laser-lesion is very high, the number of perilesional MPs increases in particular in hyperinflammatory conditions [4, 54, 55] and reflects how strongly the inflammatory cells resist the subretinal immune suppression.

The experimental animals underwent either splenectomies or sham operations 30 days before, and they were implanted with subcutaneous mini pumps containing either PBS or ANGII (at a rate of 1 µg/kg/min) 3 days prior to the laser injury. Contrary to studies in rats [56], that have been shown to be significantly more sensitive to ANGII [57], this dosage did not induce a detectable vascular leakage in angiography in our experimental mice (data not shown). CNV and perilesional subretinal macrophage infiltration was evaluated at 7 days on CD102 IBA1 double-stained flatmounts. Due to the quick downregulation of Ly6C and ATR1 in infiltrating Mos, we could not directly quantify ATR1^+^Ly6C^high^MPs. Quantifications revealed that the number of perilesional subretinal IBA1^+^ MPs was significantly increased in ANGII receiving mice compared to controls, and there was a tendency of increased CD102^+^CNV (Fig. 1F). Preventive splenectomies completely abolished the pro-inflammatory effect that ANGII exerted on the accumulation of IBA1^+^ MPs and reduced the development of CD102^+^CNV at day 7 (Fig. 1F). In terms of MP infiltration and CNV development, these lesions were not significantly different from splenectomized mice without ANGII pumps presented below (2C). Additionally, the subretinal accumulation of perilesional MPs and the size of the CNVs were comparable in laser-injured mice and mice that additionally underwent sham-operations and/or received PBS mini pumps (data not shown) indicating that neither the sham operation nor the pump implantation altered the subretinal inflammation.

Last but not least, we confirm a previous report [42] that ATR1 inhibition by daily intraperitoneal injections of the ATR1 antagonist losartan at 10 mg/kg for seven days after the laser injury significantly reduced the number of subretinal perilesional IBA^+^MPs (green) and CD102^+^CNV (red) evaluated on double labeled RPE/choroidal flatmounts compared to PBS (Fig. 1G).

Taken together our data confirm that spleMo express high levels of ATR1 and can be mobilized from the spleen by ANGII. Importantly, exogenous ANGII increased the chorio-retinal inflammation and neovascularization, and this pro-inflammatory effect was completely dependent on the presence of the spleen. These data demonstrate that the pro-inflammatory effect of an excess of ANGII on laser-induced CNV is strictly dependent on the presence of the spleen and not mediated by a direct effect of ANGII on vascular endothelial cells, resident macrophages or BMMo, which still takes place in splenectomized animals implanted with the ANGII pumps.

### Splenectomy inhibits acute subretinal inflammation independently of lymphocytes

In the early inflammatory reaction following laser-injury, MdCs represent between 20 to 50% of the subretinal MPs at lesions [6, 13, 14]. Although Tan et al. showed that splenectomies reduce laser-induced ocular inflammation [37], it remains unclear to what degree this effect might be due to an indirect effect mediated by the loss of splenic lymphocytes and to what degree subretinal lesional MdCs are derived from spleMos.

**Fig. 2:**
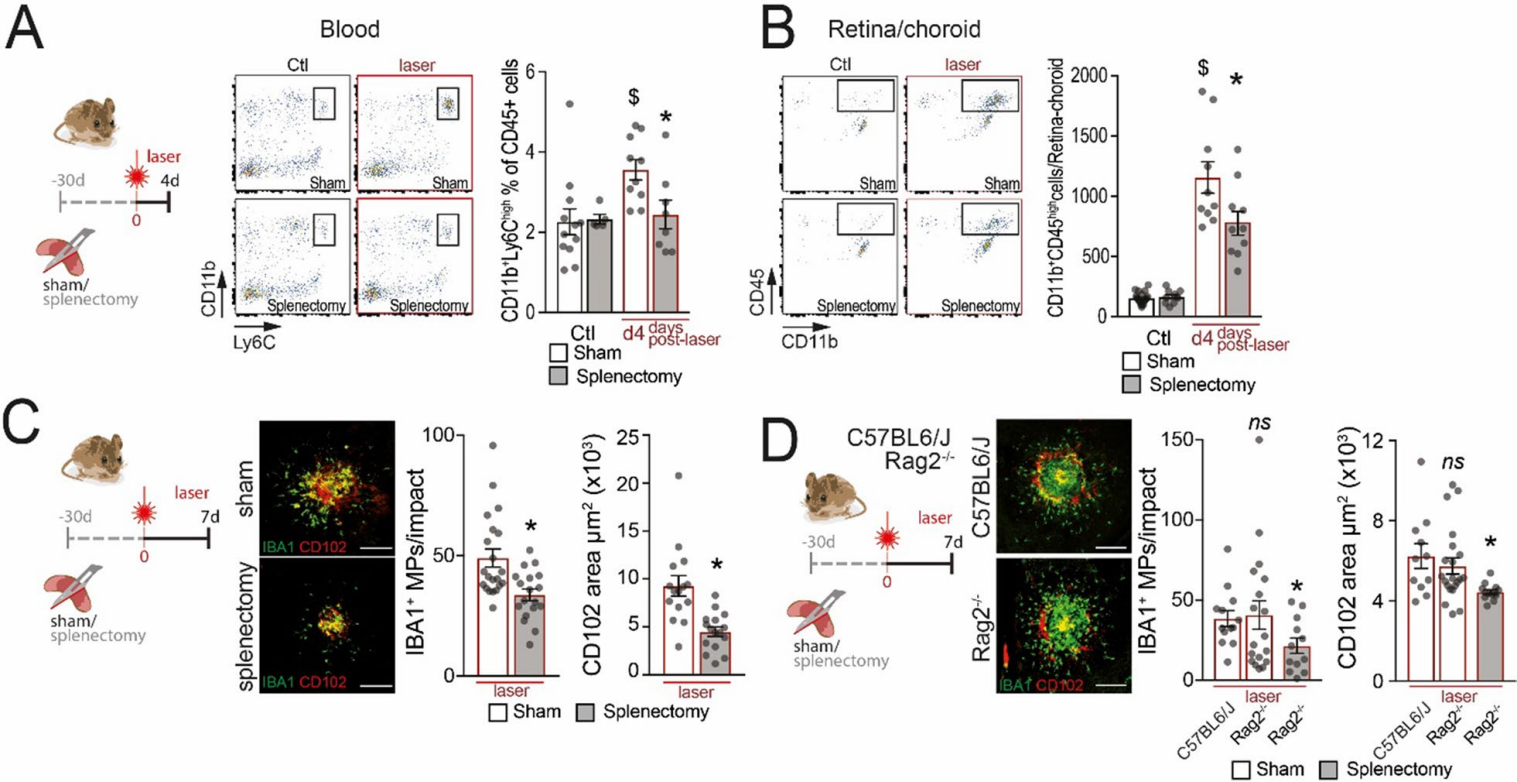
Splenectomy inhibits acute subretinal inflammation independently of lymphocytes. **A** and **B** Quantification of CD11b^+^Ly6C^high^ classical monocytes in the blood (**A**) and of CD45^high^CD11b^+^ monocyte-derived cells (MdCs) in the retina/choroid (**B**) of un-lasered controls and day4-lasered mice that have undergone splenectomies or sham-operations 30 days prior (**A** one-way ANOVA, un-lasered Ctl sham (*n* = 12) vs d4-lasered Sham (*n* = 10): $*p* = 0.0094; d4-lasered Sham (*n* = 10) vs d4-lasered Splenectomy (*n* = 8), *p = 0.046; **B** one-way ANOVA, un-lasered Ctl sham (*n* = 20) vs d4-lasered Sham (*n* = 10): ^$^*p* =  < 0.0001; d4-lasered Sham (*n* = 10) vs d4-lasered Splenectomy (*n* = 10), *p = 0.037). **C** Representative images of 7d-laser injured IBA1 (green) and CD102 (red) stained RPE flatmounts of mice that have undergone splenectomies or sham-operations 30 days prior. Quantification of IBA1^+^ MPs surrounding laser impacts and the CD102^+^ CNV area (Mann–Whitney test, Sham (*n* = 10) vs splenectomy (*n* = 18), MPs *p = 0.0056; CD102^+^ area *p < 0.0001). Scale bar = 20µm. **D** Representative images of IBA1 (green, MP marker) and CD102 (red) stained RPE flatmounts of – 30 d sham-operated wild-type and sham-operated and splenectomized *Rag2*^*−/−*^ mice 7 days after laser impact. Quantification of IBA1^+^ MPs surrounding laser impacts and CD102^+^ CNV area (*n* = 10/group; one-way ANOVA, Sham vs* Rag2*^*−/−*^ splenectomy, MPs *p = 0.033 and CD102^+^ area *p = 0.019). Scale bar = 20 µm. Ctl: control; MP: mononuclear phagocyte; RPE: retinal pigment epithelium

Our FACS analysis of the blood of control and four-day-lasered, 3-month-old, male mice that had previously undergone a sham- or splenectomy-procedure (day − 30), confirmed a previous report [37] that the laser-induced increase of circulating CD11b^+^Ly6G^−^Ly6C^high^Mos in sham-mice was abolished by the splenectomy (Fig. 2A). Accordingly, the number of CD45^high^CD11b^+^MdCs that had infiltrated the laser-injured retinal–choroidal tissue at day four were reduced (Fig. 2B). At the laser-injury site, the numbers of perilesional IBA1^+^ MPs and CD102^+^CNV at day 7 (Fig. 2C) were also both significantly reduced in splenectomized-mice compared to sham-operated animals. To evaluate whether the lack of splenic lymphocytes participated in the inhibitory effect of splenectomies, we performed splenectomies and sham operations on *Rag2*^*−/−*^ mice, that lack mature T-lymphocytes. The subretinal inflammation and CNV formation 7 days after laser injury did not differ from wild-type mice. However, preventive splenectomies significantly reduced the chorio-retinal IBA1^+^ MP inflammation and CD102^+^CNV in laser-injured *Rag2*^*−/−*^ mice at day 7 (Fig. 2D), similar to wild-type mice (Fig. 2C).

Collectively, our data confirm that splenectomy, which removes the ATR1^+^Ly6C^high^ spleMos and all other splenic cell populations, abolishes the injury-induced surge of Ly6C^high^ blood Mo and reduces the pathogenic chorio-retinal MP accumulation. Importantly, splenectomy provoked a similar reduction of subretinal perilesional MP accumulation in *Rag2*^*−/−*^ mice, suggesting that the anti-inflammatory effects of splenectomy in this model were not due to loss of splenic lymphocytes.

### Transcriptionally and functionally distinct SpleMdCs infiltrate the retina after injury

**Fig. 3:**
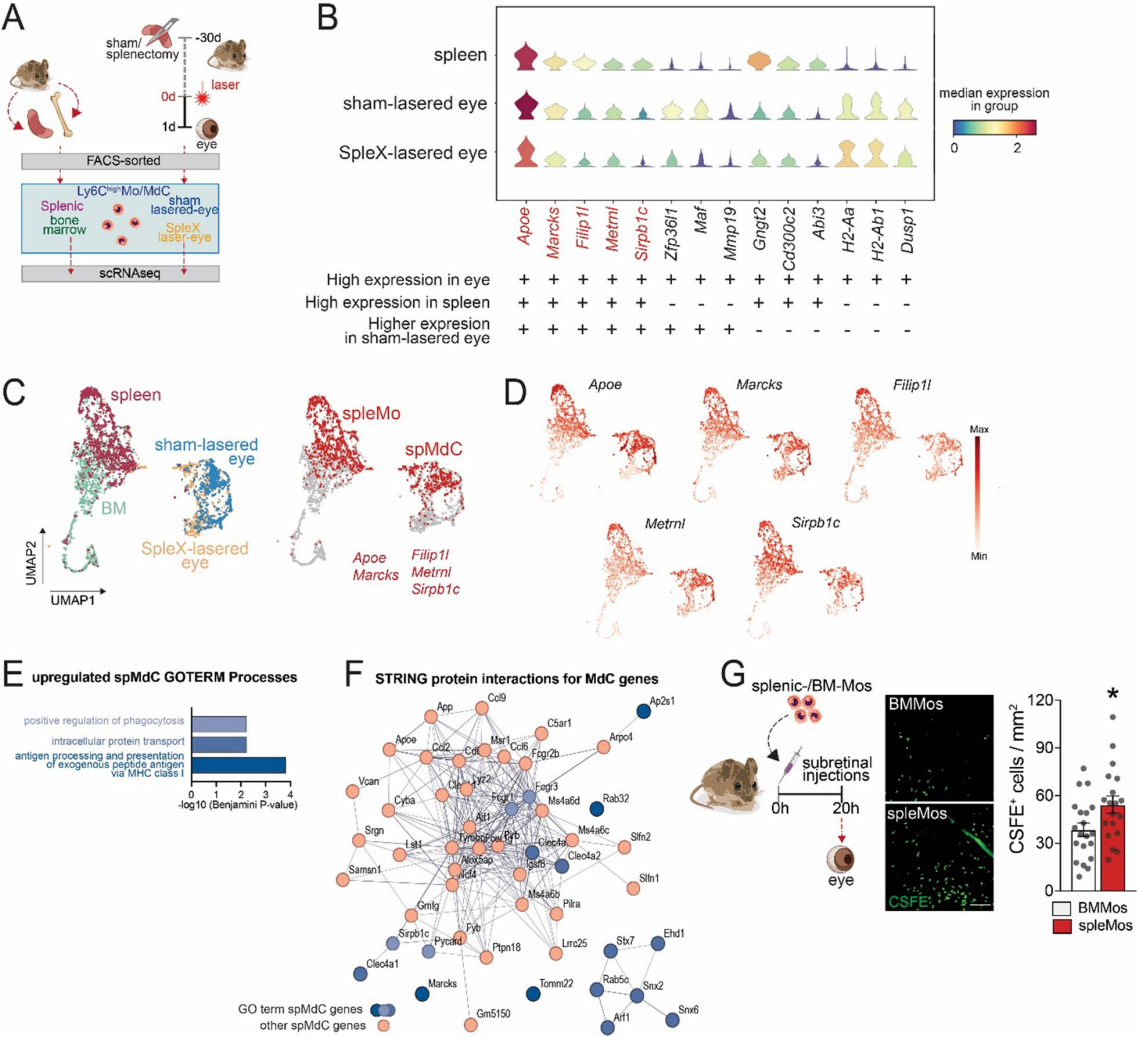
Transcriptionally and functionally distinct SpleMdCs infiltrate the retina after injury. **A** Cartoon of the experiments for the analysis of single-cell RNA sequencing. Cells were FACS-sorted from five independent mice. **B** Expression of representative upregulated genes in SpleMos compared to BMMos in CD11b^+^Ly6G^−^Ly6C^high^ cells from the spleen, CD11b^+^Ly6G^−^ cells from sham-operated lasered eyes and splenectomized lasered eyes (splenic MdC signature genes marked in red). **C** Uniform Manifold Approximation and Projection (UMAP) of scRNA-sequencing of CD11b^+^Ly6G^−^Ly6C^high^ cells from the spleen and the BM; of CD11b^+^Ly6G^−^ cells from eyes of splenectomized, 24 h-lasered mice (SpleX-lasered eyes); and from eyes of sham-operated, 24 h-lasered mice (sham-lasered eye). UMAP of the distribution of spMdCs defined by the transcriptional signature composed of *Apoe, Marcks, Filip1l, Metrnl,* and *Sirpb1c.* **D** UMAPs showing the relative expression of the five spleMdC signature genes. **E** Functional annotation of the 195 upregulated genes in spMdCs *versus* the other MPs in laser-injured eyes performed with DAVID for Gene Ontology term (GOTERM) Biological Process and showed a significant enrichment of genes associated with antigen processing, protein transport and phagocytosis pathways. **F** Known and predicted protein interaction (STRING) of the genes belonging to the significant GO term processes shown in **C** (blue dot) and the other upregulated genes (red dots) in spMdC (52/195 upregulated genes are represented). **G** Representative images of RPE flatmounts and quantification of CFSE-stained BMMos or SpleMos 24 h after adoptive transfer to the subretinal space of recipient mice (*n* = 20; Mann–Whitney test, BMMos vs SpleMos, *p = 0.0468). Scale bar = 50 µm. SpleX: splenectomy; scRNAseq: single-cell RNA sequencing; spleMo: splenic monocyte; BMMo: bone marrow monocyte MdC: monocyte-derived cell; spMdC: splenic monocyte-derived cell

To identify spleMo-derived cells (spMdCs) that infiltrate the laser-injured eyes, we next aimed to determine a transcriptional signature of these cells by single-cell RNA sequencing (scRNAseq). In order to achieve this, we employed FACS sorting to isolate CD45^+^CD11b^+^Ly6G^−^Ly6C^+^ monocytes from both the spleen (spleMo) and bone marrow (BMMo) of 3-month-old mice, as well as from chorio-retinal tissue of 3-month-old mice that had undergone laser injury one day prior. Additionally, these mice had either undergone a splenectomy (SpeX) or a sham operation 30 days before the experiment (Fig. 3A).

We hypothesized that mRNAs strongly overexpressed in spleMos compared to BMMos could be used as markers to identify infiltrating, early-differentiating MdCs that are deriving from spleMos. We analyzed the sorted cells of all groups by scRNAseq. There were 235 transcripts that exhibited significant differences (with a log2 fold change > 0.8) between spleMos and BMMos. Among these transcripts, 70 were upregulated in spleMos when compared to BMMos (Additional file 1: Table S1, numbers of analyzed cells: spleen 1116, BM 1494, sham-lasered eye 769, SpleX-lasered eye 1054; each group was pooled from 5 independent animals).

Furthermore, considering the significant transcriptome alterations during the differentiation of infiltrating Mos to MdCs in the tissue, we took an additional step of identifying transcripts that were present in at least 35% of the early differentiating Ly6C^+^ MdCs that had infiltrated the eye during the first 24 h after laser injury. As a result of this analysis, we identified 38 transcripts that differentiate spleMo (Additional file 1: Table S1). We next calculated the ratio of Ly6C^+^MdCs expressing these spleMo-marker mRNAs in the chorio-retinal tissue from sham-operated versus splenectomized mice and observed that eye-infiltrating Ly6C^+^MdCs expressing 31 out of the 38 identified spleMo-markers were more common in laser-injured mice with a spleen (Additional file 1: Table S1). By comparing gene expression of these 38 markers from the scRNAseq dataset, we determined a 5-genes signature of spMdC based on genes highly expressed in both Ly6C^+^spleMos and in Ly6C^+^MdCs from sham-operated mice (that possess a spleen): *Sirpb1c, Apoe, Filip1l, Marcks* and *Metrnl* (Fig. 3B). UMAP plots of the level of expression in cells of all groups illustrate that MPs with this 5-genes signature locate to spleMos and MPs from sham-lasered eyes compared to BMMo and MPs from splenectomized-lasered eyes (Fig. 3C and D).

The comparison of the transcripts of the infiltrating spMdCs expressing this 5-genes signature with all other MPs in laser-injured eyes allowed the further identification of 195 genes that were significantly upregulated in spMdCs and 5 that were down-regulated in infiltrating spMdCs (Additional file 2: Table S2). Functional biological process annotation analysis (Gene Ontogeny GOTERM) indicated that upregulated genes in infiltrating spMdCs are enriched in genes associated with antigen processing, protein transport and phagocytosis pathways (Fig. 3E). A network drawing using the STRING database/algorithm demonstrated that genes involved in these pathways are part of a network with the other upregulated genes in spMdC (Total 52 genes, Fig. 3F).

We next tested whether we could detect functional differences of spleMos and BMMos experimentally in the context of subretinal inflammation. CFSE-stained spleMos survived in significantly greater numbers 20 h after adoptive transfer into the subretinal space compared to CFSE^+^BMMos (counted on retinal/ RPE flatmounts, Fig. 3G), suggesting that spleMos are likely more resistant to elimination from the immunosuppressive subretinal space than BMMos, and might promote a more prolonged inflammatory reaction.

In summary, our data demonstrate that SpleMos, despite the fact that they originally derive from BMMos, acquire a transcriptionally and functionally distinct phenotype from their parent cells. Our transcriptional analysis of eye-infiltrating MdCs of sham- and splenectomized laser-injured mice suggests that infiltration of spleMos participate in the pathogenic inflammatory response of the injured eye.

Splenectomy and ATR1 inhibition curbs subretinal pathogenic inflammation in hyperinflammatory *TRE2* mice that express the AMD-risk APOE 2 isoform.

To evaluate whether spleMos played a role in other AMD models, we used targeted replacement mice that express the human AMD-risk APOE2 isoform (*TRE2* mice). We have previously shown that *TRE2* MPs express high levels of APOE and develop age-dependent chronic subretinal MP accumulation and associated photoreceptor degeneration not observed in wild-type controls raised under the same conditions [3, 6, 58, 59].

**Fig. 4:**
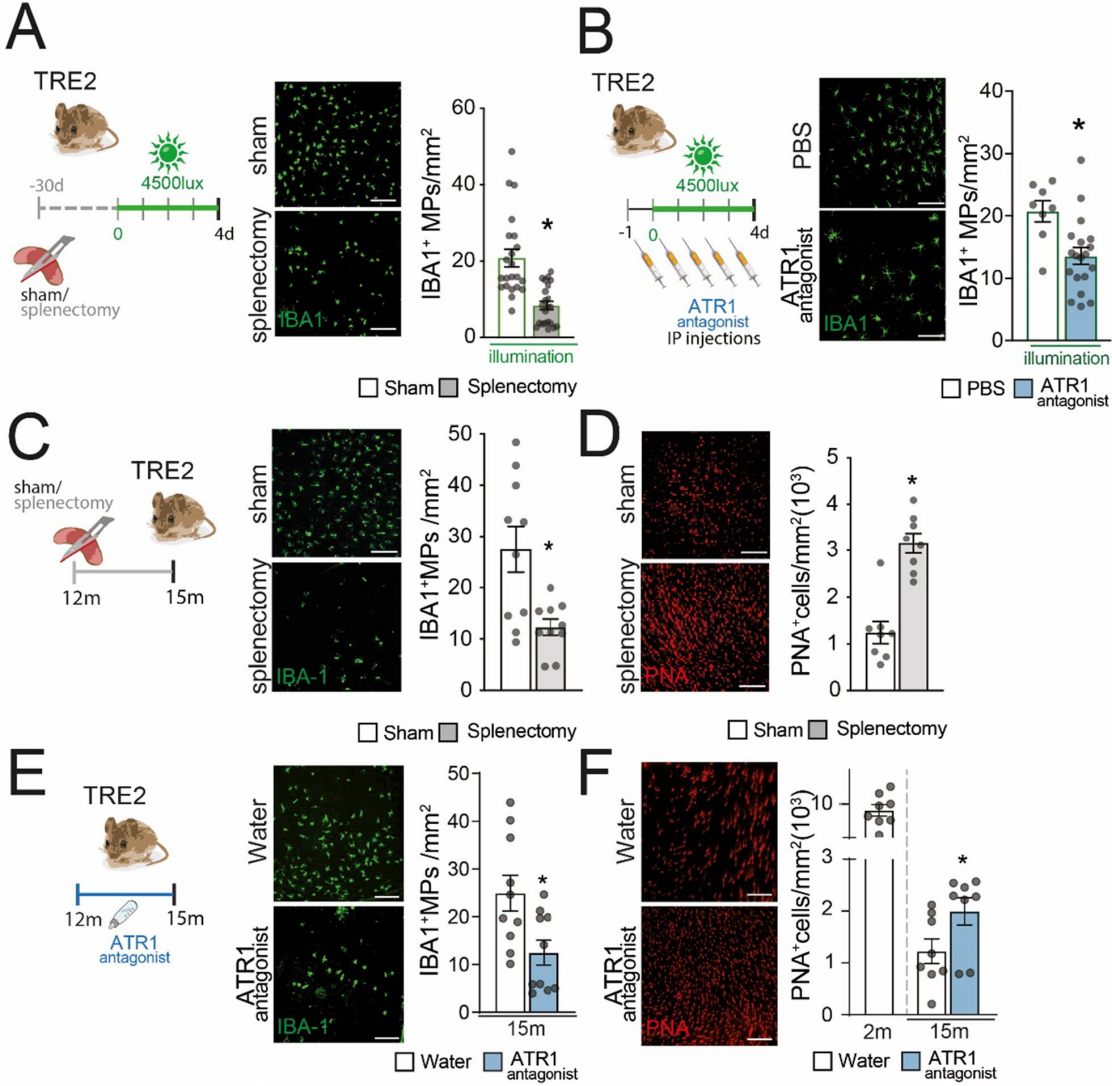
Splenectomy and ATR1 inhibition curbs subretinal pathogenic inflammation in hyperinflammatory *TRE2* mice that express the AMD-risk APOE 2 isoform. **A** Representative images of IBA1 (MP marker) stained RPE flatmounts of *TRE2* mice, after preventive (-30 days) sham or splenectomy surgeries, exposed for 4 days to 4500 lx of green light (which induces subretinal MP accumulation in these mice). Quantification of subretinal IBA1^+^ MPs (*n* = 22, Mann–Whitney test, sham vs splenectomy *p = 0.0052). Scale bar = 50µm. **B** Representative images of IBA1-stained RPE flatmounts of PBS vs losartan (10 mg/kg) treated 3-month-old male *TRE2* mice exposed for 4 days to 4500 lx of green light. Quantification of subretinal IBA1^+^ MPs in the subretinal space (Mann–Whitney test, PBS (*n* = 8) vs Losartan (*n* = 16), *p = 0.0135). Scale bar = 50µm. **C** and **D** Representative images of IBA1 (green, **C**) and PNA (red, cone marker, **D**) stained RPE and retinal flatmounts and quantification of IBA1^+^ MP (**C**) and PNA^+^ cone (**D**) density from 15-month-old *TRE2* mice that underwent sham or splenectomy surgery (at 12 months) (*n* = 10; Mann–Whitney test, Sham vs Splenectomy, MP density (**C**): *p = 0.0317, cone density (**D**): *p = 0.003). **E** and **F** Representative images of IBA1 (green, **E**) and PNA (red, **F**) stained RPE and retinal flatmounts and quantification of IBA1^+^ MP (**E**) and PNA^+^ cone (**F**) density from 15-month-old *TRE2* mice that were treated with water vs Losartan (30 mg/kg/day from 12 to 15 months) (*n* = 10; Mann–Whitney test, water vs losartan, MP density (E) *p = 0.0374; cone density (F) *p = 0.0256). Scale bar = 50 µm. ATR1: angiotensin II receptor type 1; RPE: retinal pigment epithelium; IP injection: intraperitoneal injection; MP: mononuclear phagocyte

Our experiments provide evidence that both preventive splenectomies (Fig. 4A) and daily intraperitoneal injections of the ATR1 antagonist losartan (Fig. 4B) effectively reduced the buildup of IBA1^+^ MPs, induced by 4 days of a “non-toxic” light-challenge. We quantified this reduction by examining immune-stained RPE flatmounts. The used light-intensity is not toxic in wild-type mice but triggers subretinal inflammation in these hyperinflammatory strains [6, 59].

Furthermore, splenectomies (Fig. 4C and D) and continuous addition of the ATR1 inhibitor losartan to the drinking water (Fig. 4E and F) in 12-month-old *TRE2* mice significantly reduced the age-related subretinal MP accumulation (Fig. 4C and E). In *TRE2* mice, the age-related MP accumulation is also associated with a significant decrease of the number of cones not observed in wild-type animals [3] Importantly, both treatments also increased the number of surviving cones (Fig. 4D and F) in 15-month-old *TRE2* mice, quantified on IBA1^+^(green)- and peanut agglutinin^+^(red) stained RPE- and retinal-flatmounts.

The fact that splenectomy and losartan treatment in *TRE2* mice, who carry a genetic risk variant for AMD, result in protection against retinal MP accumulation and, significantly, the associated cone degeneration, reaffirms the pivotal role of spleMos in the chronic pathogenic subretinal inflammation associated with AMD.

### AMD patients present a systemic overactivation of RAS

**Fig. 5:**
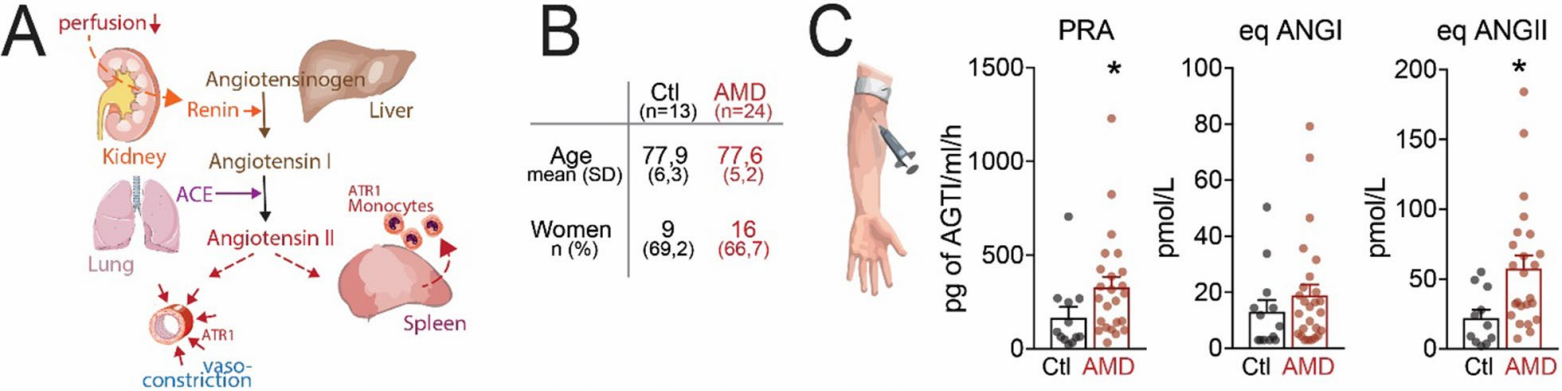
AMD patients present a systemic overactivation of RAS. **A** Schematic of the renin–angiotensin system. **B** Age and sex of the 13 controls (Ctl) and 24 AMD patients study participants. **C** Quantifications of plasma renin activity (PRA), eqANGI, and eqANGII measured after 1 h at 37 °C incubation of frozen plasma samples by LC–MS/MS (Mann–Whitney test; PRA, *p = 0.0160; eqANGII, *p = 0.0214). ATR1: angiotensin II receptor type 1; PRA: plasma renin activity; eqANG1: equilibrium angiotensin 1; eqANG2: equilibrium angiotensin II

To evaluate whether systemic RAS activation and elevated ANGII concentrations might play a role in human AMD, we measured RAS peptides in the patient’s plasma. Physiologically, the sequential hydrolysis of angiotensinogen (ANG) by renin, produced by the kidneys, generates the decapeptide angiotensin I (ANGI) that is cut by the angiotensin-converting enzyme (ACE1) of the lung to produce the very short-lived octapeptide angiotensin II (ANGII). ANGII is further processed into ANGIII and ANGIV (Fig. 5A).

We measured equilibrium (eq) ANGI and eqANGII instead of direct plasma measurements, because of the rapid speed at which RAS peptides are degraded. To do so, the samples were incubated at 37 °C and pH 7.4 for 1 h to allow ANGI and ANGII production and degradation to reach a stable equilibrium in the sample, followed by their quantification by LC–MS/MS. As the blood ANG concentration is in excess (micromolar range) compared with renin and ACE (picomolar range), these “equilibrium” concentrations are used as an approximation of the in vivo RAS activity, ANGI and ANGII concentrations [49]. Equilibrium of the RAS metabolites was rapidly reached and stable for several hours. For plasma renin activity (PRA) the samples were diluted in an ANGI stabilizing buffer containing additional ANG before a 1-h incubation at 37 °C, and the generated ANGI was determined as a measure of plasma renin activity [(ng ANGI/ml)/h] [49]. We measured RAS peptides in 24 late AMD patients (9 wet AMD patients; 15 GA patients) patients and 13 age- and sex-matched controls (Fig. 5B). Patients with established hypertension and those receiving anti-hypertensive medications were excluded from the study as these drugs interfere with the measurements.

Our measurements of AMD and control plasma samples showed a significant increase of PRA and, most importantly, eqANGII (Fig. 5C). eqANGI concentrations showed a tendency of an increase, likely because the patient’s plasma quickly converted ANGI to ANGII.

This clinical data obtained from 24 AMD patients indicate a potential link between AMD and systemic activation of the RAS. The resulting increase in circulating ANGII might trigger spleMo mobilization in human AMD similar to what we observed in the animal models of subretinal inflammation.

## Discussion

In this study, we investigated the role of spleMos among the infiltrating pathogenic MdCs observed in AMD [1, 6, 7]. Using acute injury models as well as genetic models of AMD and different approaches (ANGII injection, ATR1 blockade, splenectomy, scRNAseq profiling), we highlighted a detrimental function of ANGII-dependent mobilization of pathogenic spleMos in the chronic inflammation that drives AMD.

Experimentally, we demonstrated that Ly6C^high^spleMos localized to the subcapsular red pulp overexpressed *Agtr1a* mRNA compared to Ly6C^high^BMMos and were mobilized by ANGII, unlike Ly6C^high^BMMos (Fig. 1). Our data were consistent with findings in myocardial infarction, where ANGII participate in the recruitment of Ly6C^high^ATR1^+^spleMos [33] and suggested that ANGII contributes to subretinal MP accumulation. Indeed, we confirmed that pharmacological ATR1 inhibition reduced laser-induced subretinal inflammation and CNV [42] (Fig. 1), but as in the previous report it remained unclear whether this effect was attributable to an effect on the vasculature, on splenic monocyte mobilization, or other systemic effects.

In a series of experiments using preventive splenectomies, we demonstrated that the suppression of the spleen inhibited the laser-induced monocytosis, decreases the numbers of MdCs in the eyes and of subretinal MPs, and reduces CNV (Fig. 2). A similar anti-inflammatory effect of splenectomies on subretinal MP accumulation was also observed in light-challenged hyperinflammatory *TRE2* mice (Fig. 4). Importantly, splenectomies also completely reversed the pro-inflammatory and pro-angiogenic effect of ANGII infusions (Fig. 1), showing that the ANGII-induced aggravation of the model was fully dependent on the presence of the spleen and that eventual ANGII-induced changes of the vascular endothelium or other cell types were not sufficient to trigger the pro-inflammatory effect of ANGII in the model. Moreover, the fact that preventive splenectomies retained their anti-inflammatory effect in laser-injured *Rag2*^*−/−*^ mice that lack mature lymphocytes, demonstrated that splenic lymphocytes were not required for laser-induced, spleen-mediated, chorio/retinal inflammation (Fig. 2). Together with our data showing that spleMos were the main *Agtr1a* expressing cell type in the spleen, that ANGII infusion aggravated subretinal inflammation spleen dependently, and that pharmacological ATR1 blockage inhibited subretinal inflammation, our results strongly argued for an important pathogenic effect of Ly6C^high^ATR1^+^spleMo recruitment in the subretinal space.

To identify a transcriptomic signature of spleMo, which would allow the identification of infiltrating spleMo and early spleMo-derived cells (spMdCs) in the tissue, we next compared gene expression data from datasets of scRNAseq of Ly6C^high^ cells from spleen, BM and scRNAseq from lasered-eyes of sham-operated mice *versus* mice that had undergone a splenectomy. We were able to established a signature of five genes that identified spleMdC in the early laser-induced inflammatory reaction (Fig. 3). Among these genes, *Apoe* mRNA was one of the signature transcripts of spMdCs, which we previously showed is overexpressed in subretinal MPs in AMD lesions and in MPs of the AMD-risk APOE2 isoform-expressing transgenic *TRE2* mice [48, 59]. The identification of this signature allowed us to identify transcriptional patterns of spMdCs enriched for genes associated with antigen processing, protein transport and phagocytosis pathways (GOTERM). Similarly, pathogenic infiltrating MdCs in experimental autoimmune encephalomyelitis also exhibited the activated phagocytic phenotype at disease onset [60]. Functionally, our adoptive transfer experiments demonstrated that spleMos resisted elimination from the immune-suppressive subretinal space significantly better compared to BMMos. We previously observed a similar phenotype in MPs from *TRE2* mice that overexpress APOE compared to wild-type MPs [59], similarly to spleMo compared to BMMos. Taken together, our data demonstrate that spleMos, differ not only transcriptionally but also functionally from BMMos. SpleMos increased resistance to subretinal elimination likely further promotes pathogenic inflammation.

The laser-CNV model, commonly employed, consistently triggers subretinal inflammation and choroidal neovascularization that mirrors the characteristics of wet AMD. However, it is inherently an acute wound healing model. To evaluate if spleMos played a significant pathogenic role in a second AMD model, we used targeted replacement mice that express the human AMD-risk APOE2 isoform (*TRE2* mice). APOE2 allele carriers are at increased risk for developing late AMD [odds ratio (OR) = 1.83 for homozygote APOE2 allele carriers] [61]. We previously showed that *TRE2* mice develop age-dependent chronic subretinal MP accumulation and associated photoreceptor degeneration, similar to human disease [3, 6, 58, 59].

Our findings demonstrated that in *TRE2* mice, both splenectomy and losartan treatment were effective in preventing the accumulation of subretinal MPs. Crucially, the reduction in chronic MP buildup in aged mice through both interventions also led to a significant decrease in associated cone degeneration. This underscored the critical role of spleMos in the chronic pathogenic subretinal inflammation associated with the AMD-risk APOE2 isoform.

In a prior study, Tan et al*.* showed that when wild-type mice underwent splenectomy, they exhibited a decrease in both CNV and Ly6C^high^Mos infiltration in the eye [37]. However, their research did not elucidate the extent to which ANGII plays a role in this phenomenon. Moreover, it remains uncertain whether spleMos directly contributed to the subretinal infiltrate or if the splenectomy had an indirect impact [37]. Nagai et al*.* demonstrated a favorable outcome with ATR1 inhibition in both a laser-induced CNV model and a high-fat diet model [42, 43]. But they ascribed this positive effect to the local inhibition of ATR1 on choroidal vessels and lipid-loaded choroidal macrophages independently of the spleen [42, 43]. Taken together, our results suggest an important role for angiotensin dependent mobilization of spleMos in the pathogenesis of AMD: ANGII pumps mobilized Ly6C^high^Mos from the spleen and only increased subretinal inflammation and CNV when the spleen was present. These results ruled out an important role of local and vascular effect ATR1 activation in the CNV model. Furthermore, our results expand previously published reports by using the *TRE2* mice that mirror precisely an AMD genetic risk factor.

To further investigate whether ANGII-induced spleMo might play a role in the human disease, we set out to detect increase in RAS activity. Since the RAS peptides, but not angiotensinogen, renin, or ACE, are quickly degraded, we here first measured the ability of patient- and control-plasma to generate ANGI and ANGII as an approximation for the subject’s systemic RAS activation. Our experiments showed a significant increase in PRA and eqANGII in patients, compared to age-matched controls (Fig. 5). Although we excluded patients and control subjects who are on anti-hypertensive medicine as the medication interferes with the plasma peptide generation and their measurements, we do not exclude that increased RAS activity might be in part due to hypertension, which is associated with AMD [39]. It could also be increased to stabilize blood pressure in situations such as dehydration, which is often observed in the elderly.

**Fig. 6:**
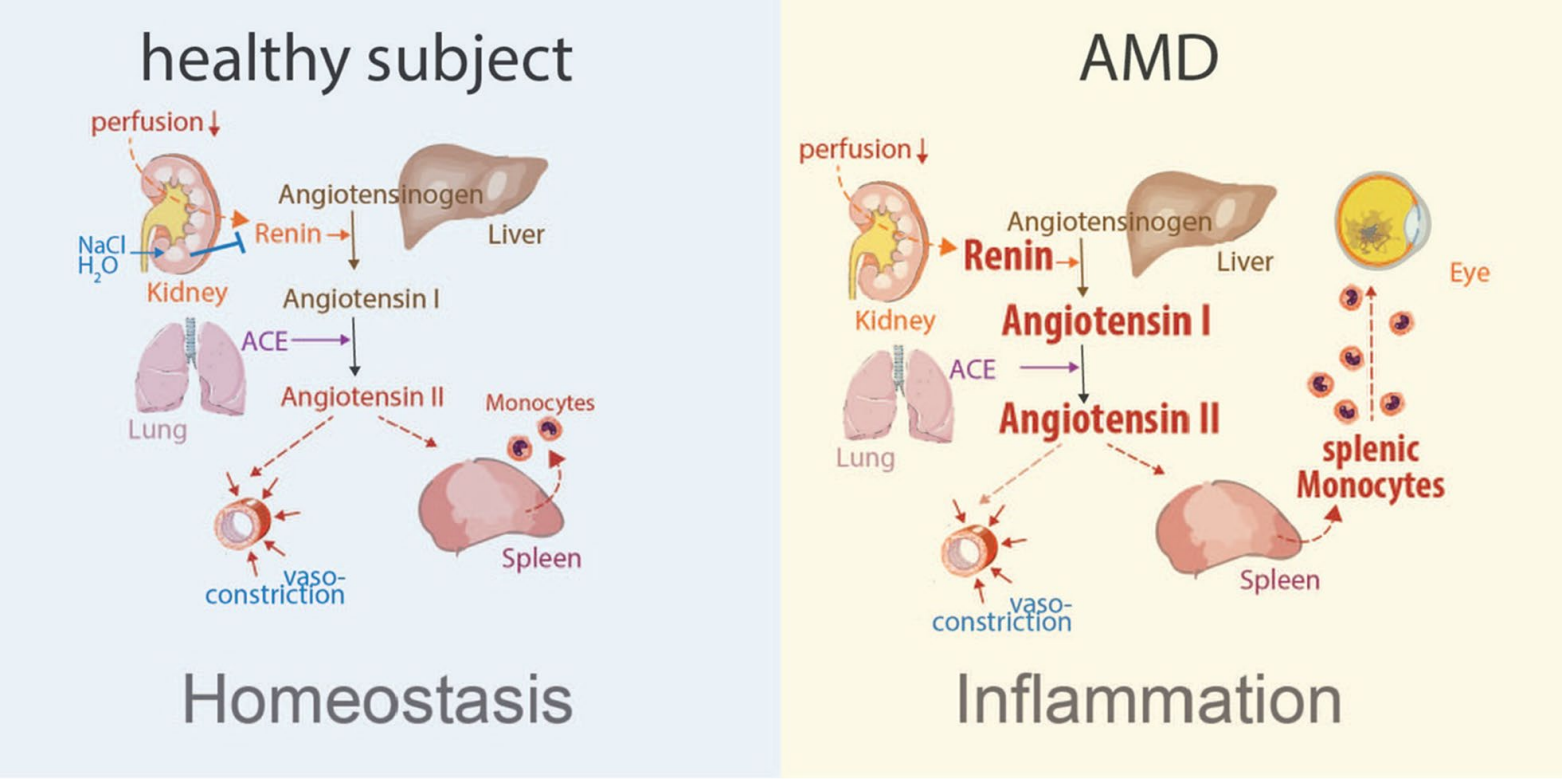
Schematic representation of the involvement of splenic monocytes in pathogenic inflammation in AMD. Physiologically the renin–angiotensin system (RAS) is a master regulator to maintain blood pressure. We here show that the RAS activation and angiotensin II elevation mobilizes splenic monocytes that participate in and aggravate retinal inflammation and associated neovascularization and photoreceptor degeneration. Increased angiotensin II levels in patients compared to controls suggest that similar mechanisms take place in AMD

Remarkably, a recent survey of 3,023 hypertensive patients revealed that the duration of RAS inhibitor treatment, a factor previously overlooked in past studies, is inversely linked to the prevalence of AMD. This finding underscores the potential significance of RAS activation in the pathogenesis of AMD. Elevated systemic ANGII in AMD patients could lead to altered chorio-retinal perfusion, but also to the mobilization of pathogenic spleMos. The exaggerated recruitment of spleMos to the eye in AMD might push the balance to a more destructive inflammation (Fig. 6). AMD patients, particularly with high eqANGII, might benefit from ATR1 antagonists or other means to reduce spleMo recruitment to the eye.

### Supplementary Information

**Additional file 1: Table S1.** Fold Changes (FC) of the 70 genes upregulated between SplMo and BMMo in scRNAseq experiment. The frequency of Ly6C^+^Mos in the spleen, in sham Eye and SpleX eye represents the percentage of cells with an expression level > 0 for the selected gene in scRNAseq among the total of Ly6C^+^Mos in each organ. The Ratio %age Sham vs SpleX eye is the ratio of frequency of Ly6C^+^Mos in Sham to frequency of Ly6C^+^Mos in SpleX eye. In red, genes of the spleMdC signature.

**Additional file 2: Table S2.** 195 up-regulated genes and 5 down-regulated genes in MPs expressing the spleMdC signature compared to all other MPs in laser-injured eyes.

## Data Availability

Further information and requests for resources, datasets and processing scripts should be directed to and will be fulfilled by the corresponding authors.
